# Comparison of Mid‐term Survivorship and Clinical Outcomes between Bipolar Hemiarthroplasty and Total Hip Arthroplasty with Cementless Stem: A Multicenter Retrospective Study

**DOI:** 10.1111/os.12440

**Published:** 2019-04-12

**Authors:** Chen‐Chiang Lin, Chang‐Chen Yang, Tzai‐Chiu Yu

**Affiliations:** ^1^ Department of Orthopaedic Surgery National Taiwan University Hospital (Yulin Branch) Yulin Taiwan; ^2^ Department of Orthopaedic Surgery Tzu‐Chi Hospital Dalin Branch Chiayi Taiwan; ^3^ Department of Orthopaedic Surgery Taipei Tzu‐Chi Hospital New Taipei City Taiwan

**Keywords:** Bipolar hemiarthroplasty, Cementless hip stem, Oxford hip score, Survivorship analysis, Total hip arthroplasty

## Abstract

**Objectives:**

To compare the clinical outcome between bipolar hemiarthroplasty (BHA) and total hip arthroplasty (THA) using a U2 HA cementless hip stem, and the results of elderly femoral neck fracture patients who underwent BHA with a cementless hip stem.

**Methods:**

A multicenter retrospective study enrolled 96 BHA and 115 THA cases using U2 HA cementless hip stems with mean age (BHA: 67.9 years; THA: 64.1 years), body height (BHA: 160.4 cm; THA: 160.7 cm) and weight (BHA: 62.7 kg; THA: 64.5 kg) recorded. Mean follow‐up durations were, respectively, 7.1 (BHA) and 7.8 (THA) years. Survivorship analyses and Oxford hip scores were compared.

**Results:**

Both the BHA and the THA groups revealed high survival rates at 5‐year (100%) and 10‐year (100.0% and 90.1%) follow‐up. The THA group achieved better joint performance and pain relief. The cementless HA stems had survived perfectly for 10 years for elderly femoral neck fracture patients following BHA.

**Conclusions:**

The U2 HA cementless hip stem provides an effective solution for both BHA and THA surgeries, and for elderly femoral neck fracture patients undergoing BHA. According to the findings of the current study, THA may be inadequate for addressing avascular necrosis, and pain control is a considerable concern for patients who have undergone BHA.

## Introduction

Hip joint arthroplasty is the last resort for treating articulating surface disorders (i.e. osteoarthritis, rheumatoid arthritis, and avascular necrosis) or joint structure damage (i.e. femoral neck fracture, subcapital fracture, and intracapsular fracture), which cannot be repaired using conservative treatment or trauma devices. The procedure for hip joint arthroplasty can be either a total hip arthroplasty (THA) or a hemiarthroplasty, referring to the severity of hip joint damage. Hemiarthroplasty is applied for patients suffering from damage of the femoral head or proximal femur with the acetabulum remaining intact. The total hip arthroplasty involves replacing both acetabular and femoral components to achieve a full artificial articulation. Previous studies have demonstrated that better postoperative outcome was attained for patients who underwent THA than those who underwent hemiarthroplasty^1^, ^2^, but the risk of joint dislocation after THA surgery may be higher because of the structural characteristics^3^, ^4^, ^5^, ^6^. Advantages of hip hemiarthroplasty include lower risk of joint dislocation, less surgical complexity, reduced operation time and intraoperative blood loss, and decreased initial costs^7^, ^8^; however, acetabulum erosion and groin pain after surgery have puzzled surgeons and troubled patients greatly^9^, ^10^.

Both THA and hip hemiarthroplasty have been discussed or compared under various indications for their benefits in clinical practice. However, it is regrettable that uncontrolled implants, which may increase the variables in comparisons, have not been well excluded. Therefore, the current multicenter retrospective investigation, which recruited patients who underwent THA or hip hemiarthroplasty with an identical cementless femoral implant, was designed for comparison. Besides survivorship analysis, the Oxford hip score^11^, ^12^ has also been applied to evaluate patients’ daily performance and complaints after surgery through telephone questionnaires^13^. Concerns regarding postoperative complications and possible solutions to enhance the quality of surgery were also discussed.

Another issue is whether using a cementless stem is applicable for elderly patients (aged over 70 years) with femoral neck fractures. Previously, treatment with cemented bipolar hemiarthroplasty (BHA) was the standard policy of the National Health Insurance in Taiwan for femoral neck fractures in elderly patients. Studies have suggested using cemented stems as well in view of pain relief, functional outcome, and comparatively lower incidence of periprosthetic fractures^14^, ^15^. With the advancement of surface coating techniques, promising clinical results have also been revealed, especially for stems with hydroxyapatite coating^16^, ^17^. Therefore, patients aged over 70 treated with BHA for femoral neck fractures using cementless stems have been reviewed as well.

## Materials and Methods

### *Patient Selection*

**Figure 1:**
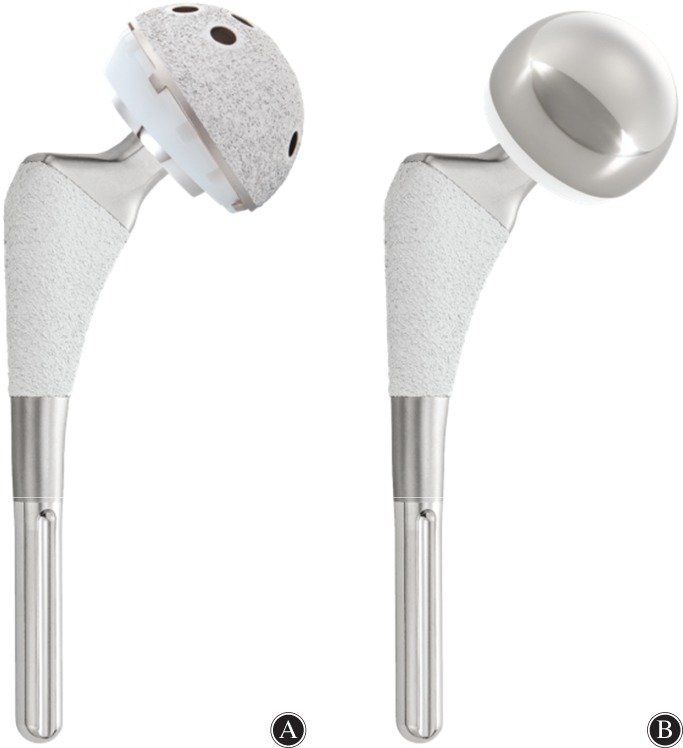
The U2 HA hip stem (United Orthopedic Corporation, Taiwan) for (A) total hip arthroplasty and (B) bipolar hemiarthroplasty.

Three hospitals in Taiwan (Tzu‐Chi Hospital, Taipei and Dalin Branches, and National Taiwan University Hospital, Yulin Branch) participated in this multicenter retrospective study, with respective permission granted from the Ethical Committees (IRB approval numbers: A10501003 and 201604059RINA). To isolate the bearing surface of acetabulum as the sole variable, 211 patients who received a hydroxyapatite (HA) coated cementless hip stem (U2 HA hip Stem, United Orthopedic Corporation, Taiwan) implantation between May of 2000 and December of 2012 were enrolled. A total of 115 patients underwent THA (U2 Acetabulum System, United Orthopedic Corporation, Taiwan) with an HA coated cementless cup and ultra‐high molecular weight polyethylene (UHMWPE) liner (Fig. 1A); 96 patients underwent BHA (U1 Bipolar Hip System, United Orthopedic Corporation, Taiwan, Fig. 1B). Femoral heads utilized in THA were made either of cobalt–chrome alloy or of ceramics. For BHA cases, the articulation components were composed of a bipolar cap, UHMWPE liner, and a cobalt–chrome alloy femoral head.

### *Data Acquisition*

Information including patients’ age, gender, height, weight, body mass index (BMI), diagnosed cause of primary surgery, and cause of revision was recorded. The BHA group consisted of 44 men and 52 women, while 57 men and 58 women were included in the THA group. The mean age of patients was 67.9 and 64.1 years for BHA and THA groups, respectively. The average duration of follow‐up for BHA and THA groups was, respectively, 7.1 (3.6–12.3) and 7.8 (3.6–16.3) years. General information for patients is listed in Table 1.

**Table 1:** General information of patients enrolled in the current study

Index	BHA	THA	*P*‐value
Gender			
Male	44	57	0.59
Female	52	58
Age (years)			
30–40	1	7	0.14
41–50	14	16
51–60	13	21
61–70	22	27
71–80	26	30
81–90	20	12
91–	0	2
Average	67.9 ± 13.5	64.1 ± 14.7	
Other information (mean ± SD)			
Height (cm)	160.4 ± 8.1	160.7 ± 7.9	0.41
Weight (kg)	62.7 ± 11.3	64.9 ± 12.9	0.12
BMI (kg/m^2^)	24.3 ± 3.8	25.1 ± 4.3	0.08

BHA, bipolar hemiarthroplasty; THA, total hip arthroplasty.

### *Survivorship Analysis*

Survivorship of both BHA and THA groups was analyzed using the Kaplan–Meier survivorship curve method^18^, while the record of revision due to infection was excluded. Period of implant survivorship was defined by the duration from the primary surgery to the date of revision. If there was no revision surgery required, the date of telephone questionnaires completed would then be the end of the survivorship record, instead of the end of implant survivorship.

### *Oxford Hip Score*

The Oxford hip score is a short 12‐item questionnaire^11^, ^12^ filled in by telephone interview in the current study. The Oxford hip score is generally used to evaluate the hip joint function and pain of patients.

The score for each question ranged from 0 (most severe or totally incapable) to 4 points (problem free or easy to achieve) for each of the 12 items. Higher Oxford hip score indicates greater capability of patients in hip joint function. Scores were compared between the THA and BHA groups to identify the major influences on performance. The scores were presented as excellent (more than 41), good (34 to 41), fair (27 to 33), and poor (less than 27), and can be compared to Harris hip scores referring to the categorization principle suggested by Kalairajah *et al*.^19^.

### *Statistical Analyses*

Statistical differences in gender and age between the BHA and the THA groups were determined by χ^2^‐tests. For body height, weight, BMI, and the total score and items in the Oxford hip score, independent *t*‐tests were conducted to identify whether significant differences existed between the BHA and the THA groups. The T‐score method^20^ was applied to compare the population distribution for the Oxford hip scores, with further focus on the specific disease avascular necrosis.

## Results

### *General Information*

General information on patients in both BHA and THA groups, including gender, age, body height, weight, and calculated BMI, are listed in Table 1. No significant differences were found.

### *Diagnosed Cause of Surgery and Cause of Revision*

In the BHA group, the diagnosed causes of primary hip arthroplasties were avascular necrosis (33 of 96 hips), fracture (60 of 96 hips), and others (3 of 96 hips). In the THA group, surgeries were conducted for osteoarthritis (80 of 115 hips), avascular necrosis (26 of 115 hips), fracture (4 of 115 hips), hip dysplasia (2 of 115 hips), rheumatoid arthritis (1 of 115 hips), and others (2 of 115 hips) (Table 2). Complications and complaints by patients, such as pain, muscle weakness, claudication, joint dislocation, and leg length discrepancy, are summarized in Table 3. In the BHA group, 1 patient underwent revision surgery due to infection; in the THA group, 9 patients underwent revision surgeries because of cup loosening (7 hips) and infection (2 hips). Table 4 presents information for cases of implant revisions, including the age, gender, diagnoses for primary hip arthroplasty, survival period, and etiology for revision.

**Table 2:** Diagnosed cause of primary hip arthroplasty and the corresponding number of revision surgery and revision rate

Diagnosed cause of surgery	BHA			THA		
	Primary case(s)	Revision case(s)	Revision rate (%)	Primary case(s)	Revision case(s)	Revision rate (%)
Osteoarthritis	0	0	‐	80	3	3.8
Avascular necrosis	33	0	0	26	5	19.2
Fracture	60	1	1.7	4	0	0
Hip dysplasia	0	0	‐	2	1	50.0
Rheumatoid arthritis	0	0	‐	1	0	0
Others	3	0	0	2	0	0

BHA, bipolar hemiarthroplasty; THA, total hip arthroplasty.

**Table 3:** Postoperative complications/complaints for patients in the BHA and the THA groups

Complication / complaints	BHA	THA
None	59	59
Pain	30	27
Muscle weakness	1	2
Claudication	5	21
Joint dislocation	0	9
Leg length discrepancy	1	12

BHA, bipolar hemiarthroplasty; THA, total hip arthroplasty.

**Table 4:** Information for patients who received revision surgery

Age (years)	Gender	Diagnosis	Arthroplasty type	Survival period (months)	Cause of revision
84	Female	Fracture	BHA	6	Infection
80	Male	Osteoarthritis	THA	117	Infection
68	Female	Osteoarthritis	THA	138	Cup loosening
41	Male	Osteoarthritis	THA	131	Cup loosening
41	Female	Hip Dysplasia	THA	94	Cup loosening
72	Female	Avascular necrosis	THA	79	Cup loosening
59	Male	Avascular necrosis	THA	95	Cup loosening
57	Male	Avascular necrosis	THA	117	Cup loosening
67	Female	Avascular necrosis	THA	154	Cup loosening
38	Male	Avascular necrosis	THA	11	Infection

BHA, bipolar hemiarthroplasty; THA, total hip arthroplasty.

### *Survivorship Analysis*

**Figure 2:**
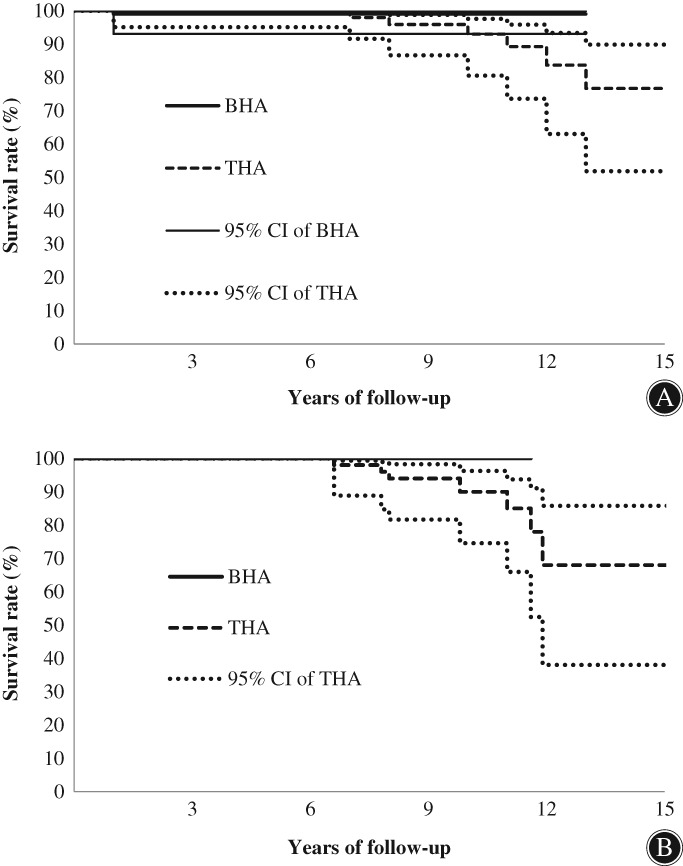
The Kaplan–Meier survival curves of survivorship to revision in full follow‐up period: (A) revision due to any reason and (B) revisions that excluded infections (solid line: bipolar hemiarthroplasty [BHA]; dotted line: total hip arthroplasty [THA]). CI, confidence interval.

The Kaplan–Meier survivorship curves of both BHA and THA groups in the current study are shown in Fig. 2A, while the graph in Fig. 2B excludes the revision cases due to infection. The survival rate fell within the first 2 years after surgery (Fig. 2A) and remained stable to the end of follow‐up in the BHA group. Great initial survivorship was exhibited in the THA group compared to the BHA group, but a decline in survivorship after 6.6 years of primary surgery was revealed. The survival rates at the 5th and 10th year for the THA group were, respectively, 100.0% and 90.1% (Fig. 2B), while that for the BHA group remained at 100.0% at the aforementioned two checkpoints (Fig. 2B).

### *Oxford Hip Score*

Mean Oxford hip scores for the BHA and THA groups were, respectively, 37.5 ± 9.6 and 44.6 ± 4.6, with significant statistical difference (*P* < 0.001). Based on the study of Kalairajah *et al.* 19, the Oxford hip scores were redistributed for the BHA (excellent: 46; good: 18; fair: 17; poor: 15) and the THA (excellent: 97; good: 13; fair: 4; poor: 1) groups (Table 5). In addition, the average scores for the 12 items were compared to determine whether statistically significant differences could be identified (Table 6, with abbreviated descriptions for each of the 12 items in the Oxford hip score questionnaire). All items showed statistically significant differences, except for the “limping” item.

**Table 5:** Translated Oxford hip score grading and the average Oxford hip score

Oxford hip score grading	BHA	THA
Excellent (42–48)	46	97
Good (34–41)	18	13
Fair (27–33)	17	4
Poor (0–26)	15	1
Average Oxford hip score	37.5 ± 9.6	44.6 ± 4.6

BHA, bipolar hemiarthroplasty; THA, total hip arthroplasty.

**Table 6:** Comparisons of 12 questionnaire items of Oxford hip score (mean ± SD)

Brief description	BHA	THA	*P*‐value
General pain	3.4 ± 0.8	3.7 ± 0.5	<0.001
Pain at sleep	3.5 ± 0.7	3.9 ± 0.3	<0.001
Sudden severe pain	3.6 ± 0.7	3.8 ± 0.4	0.03
Limping	3.3 ± 1.1	3.4 ± 0.9	0.26
Walking	3.9 ± 0.5	3.7 ± 0.7	0.013
Stair climbing	2.3 ± 1.6	3.5 ± 0.8	<0.001
Wearing socks	2.9 ± 1.2	3.7 ± 0.8	<0.001
Sit to stand	3.6 ± 0.8	3.9 ± 0.3	<0.001
In/out transportation	2.9 ± 1.2	3.7 ± 0.6	<0.001
Bathing	3.1 ± 1.2	3.9 ± 0.5	<0.001
Shopping alone	2.6 ± 1.5	3.8 ± 0.7	<0.001
Work interference	2.4 ± 1.6	3.6 ± 0.8	<0.001

BHA, bipolar hemiarthroplasty; THA, total hip arthroplasty.

### *Patients Aged over 70 years with Femoral Neck Fracture Treated by Bipolar Hemiarthroplasty*

Among the 60 femoral neck fracture patients in the BHA group, 43 patients were over 70 years old. Apart from 1 of the 43 patients who suffered from infection and underwent revision surgery, no stem loosening was observed, with a mean follow‐up period of 6.5 ± 2.3 years. The mean Oxford hip score was 31.5 ± 9.3 (excellent: 10; good: 31; fair: 2).

### *Comparison with T‐Score Method*

Tables 7 and 8 show the calculated T‐scores and the corresponding raw Oxford hip scores for all patients, the BHA group, and the THA group, with all causes of surgery and specifically with avascular necrosis. The T‐score of 50 represents the average population score, while that of 40 or 60 is one standard deviation under or over the average score. A T‐score of 30 or 70 means two standard deviations under or over the average score. Both tables reveal an obvious shift to greater scores, reflecting the positive effect of pain relief and functional repair after hip joint arthroplasty, especially for the THA group.

**Table 7:** T‐scores and raw scores (points) for the Oxford hip scores of all patients

T‐scores	Overall	BHA	THA
30	25.2	18.4	35.5
40	33.3	28	40.1
50	41.4	37.5	44.6
60	48	47	48
70	48	48	48

**Table 8:** T‐scores and raw scores (points) for the Oxford hip scores of patients with avascular necrosis

T‐scores	Overall	BHA	THA
30	34	32	37.2
40	39	37.7	41
50	44.1	43.5	44.9
60	48	48	48
70	48	48	48

## Discussion

Many retrospective studies comparing BHA and THA have been reported. When focusing on the difference between the two surgical treatments, variables such as the type of acetabulum shell, liner, and femoral stem should be unified to avoid possible bias. Previously, identical cemented hip stems for BHA and THA were used for comparison, and generally supported that THA provided a better outcome^1^, ^5^. However, the use of cementless stems has not been well controlled in related studies^4^, ^8^, ^10^, ^21^, ^22^, while the clinical outcomes and suggestions from studies were diverged. In the current study, the multicenter retrospective analysis with mean follow‐up duration of 7 years (minimum 3.5 years) for both BHA and THA groups has been conducted using the same cementless femoral stem. The results of the current study may provide a more objective viewpoint for understanding these two strategies for hip joint arthroplasty when a cementless femoral stem is applied.

Using a U2 HA hip stem in either BHA or THA surgery is the inclusion criteria in the current study. The main purpose is to reduce the variability in stem selection. In addition, its unique matrix distribution (multiple proximal and distal size combinations) of specification can achieve better fitness at both proximal and distal regions of the femoral canal^23^. With its surface HA coating in the proximal region, it is expected to enhance the long‐term stability and survival of the femoral stem so that the current study can focus on the comparison between the clinical outcome of BHA and THA groups. In the current study, no femoral stem loosening was observed in the full period of follow‐up. This successful selection of the femoral component enabled the current study to focus on the clinical outcome comparisons between BHA and THA groups.

It has been recognized that the short‐term clinical outcome is more favorable for THA than BHA^1^, ^2^. Effective pain relief can be achieved by replacing both femoral and acetabular components so that there is no perception of joint contact during articulation. Major concerns for BHA are that postoperative groin/gluteus pain and possible acetabulum protrusion may lead to surgery failure and that further revision surgeries may be required^9^, ^10^. In the current study, 31.3% (30 of 96 patients) of patients in the BHA group complained of postoperative pain. The Oxford hip scores also revealed that significant influences of pain on hip performances (Table 6) in the BHA group may be the major cause of its lower postoperative satisfaction (Table 5). The *t*‐distribution of the Oxford hip score is more negatively skewed in the THA group compared to the BHA group (Table 7). Isobe *et al*. indicate that the required time for walking speed recovery is significantly longer for patients who undergo BHA compared to those who undergo THA^21^, as well as that the need of walking aids is significantly higher among patients who have undergone hip hemiarthroplasties^22^. However, clinical investigations have also reported great long‐term survival rates for patients who have undergone BHA^24^, ^25^. Through improvements in hemiarthroplasty techniques and moving from unipolar to bipolar prosthetic design, problems of groin pain and acetabulum protrusion have been reduced^26^. Kiekens *et al*. report in their clinical series that the young patient population (mean age of 39.6 years) did not show any obvious acetabular erosion and pain due to bipolar hip prostheses implantation^24^, while another study demonstrated a 10‐year survival rate of 92.3% for patients with femoral head osteonecrosis treated by bipolar hip arthroplasties, without radiographic signs of implant loosening and osteolysis^25^. Previous biomechanical studies confirmed that an oversized prosthetic femoral head in hip hemiarthroplasty would severely increase the stress at the periacetabulum and medial wall regions of the pelvis, while a prosthetic femoral head that is too small could cause erosion, polar stress, and head migration^27^, ^28^. Inadequate size of the prosthetic femoral head for BHA may be the major cause of pain among the aforementioned problems. Therefore, careful preoperative planning and precise intraoperative measurement for the optimal size of the femoral head prosthesis is the key to enhancing the quality of BHA.

Since hip arthroplasty has been recognized as a mature surgical technique for the treatment of hip disorders, a high survival rate after surgery has been revealed in previous research^29^. Keating *et al*. reported a similar but slightly better survival rate for THA compared to BHA over a 24‐month follow‐up period^1^. In the current study, the THA group had a perfect survival rate at 6.6 years (100.0%), as did the BHA group, but this gradually declined thereafter (Fig. 2B). Although there was greater short‐term performance and survival rate for the THA group, progressive implant polyethylene wear is inevitable. The BHA group in the current study revealed great survivorship (100.0%), except for 1 revision case which was excluded from the calculation of survivorship due to infection within 6 months postoperatively. Correct implant selection and adequate surgical operation to the indications are the keys to long‐term survivorship of joint arthroplasty. This result is encouraging for the usage of BHA, but we still need to be mindful that the low revision rate may have resulted from the reduced activity level due to postoperative pain. Further studies and implant designs should be focused on how to alleviate the pain caused by BHA to enhance patients’ quality of life and functional performance. With the extended follow‐up period over 10 years, a remarkable decrease in survivorship of the THA group was revealed. Due to the sample sizes for both BHA and THA groups being reduced to under 20% of the initial sample sizes by the 10th year of follow up, the survivorship curves for both groups were discussed for the first 10 years for a fair comparison.

Leg length discrepancy is a challenge for total hip arthroplasty. Because replacements of both femoral and acetabular components are conducted in THA, difficulty in correcting limb length can be an issue because hip joint center and soft tissue tension must be maintained to achieve adequate hip biomechanics. However, in clinical practice problems with pain relief and joint stability generally arise prior to the leg length equalization^30^. A wide range for the incidence of postoperative length discrepancy has been reported, from 1% to 50%, after THA^31^, ^32^, ^33^, ^34^. In the current study, 10.4% of patients (12 of 115 hips) suffered from leg length discrepancy in the THA group, while only 1.0% of patients (1 of 96 hips) in the BHA group encountered this problem. Due to the limited accuracy of preoperative template evaluation^35^, ^36^, intraoperative techniques for better correction will be essential to reduce the problem of length discrepancy^37^.

Another issue is the use of THA in the treatment of avascular necrosis. Although comparable short‐term clinical outcomes for THA in the treatment of patients with femoral head osteonecrosis compared to those with osteoarthritis was suggested by Mont *et al*.^38^, several studies report poorer results of THA for treatment of femoral head osteonecrosis compared to osteoarthritis^39^, ^40^. The current study has revealed a relatively higher revision rate in the treatment of avascular necrosis in the THA group (19.2%, 5 of 26 hips with avascular necrosis treated by THA) compared to the BHA group (no revision case). Specifically, 4 of the 5 patients in the THA group for treatment of avascular necrosis underwent revisions due to cup loosening. A possible reason is that the affected acetabular side has not been carefully evaluated. Craiovan *et al*. report in their clinical study that poor periacetabular bone quality (lower bone mineral density), against the femoral head with osteonecorsis, is responsible for premature loosening of the acetabular cup in total hip arthroplasty^41^. This problem may cause the acetabular cup to migrate to an unfavorable location or orientation that causes a poor wear pattern of the liner. Surgeons need to pay attention to the bone quality of the periacetabular region while preparing the acetabular space. However, better results for Oxford hip score were observed for the THA group than the BHA group, while the T‐score evaluation represented better outcome for THA patients who underwent surgeries due to avascular necrosis than those who underwent BHA surgeries (Table 8). The THA surgery replaces the diseased acetabulum using an acetabular cup and liner to restore the articulation of the hip joint. Direct pain relief may be received. For the BHA surgery, the acetabulum is preserved for joint articulation. The sensation of a foreign body or possible incongruence between the bipolar head and acetabulum may lead to discomfort and pain. Because the survival rate itself does not reflect the whole picture of postoperative performance, a careful cross‐verification between clinical evaluation results such as survivorship and patient report outcome measures (PROM) should be undertaken. As for the influence of hip dysplasia on the risk of revision in the THA group, the sample size is too small for an objective evaluation so this it is not discussed in the current study.

For patients aged over 70 years, hemiarthroplasty with cemented stem is suggested for treatment of femoral neck fractures. Evidence of greater clinical outcomes, in relation to pain relief, ability to walk and the use of walking aids, and daily activities, have been reported^14^. Lower risk of periprosthetic fracture after cemented stem implantation compared to cementless stem is also an issue when considering the surgical option^15^. Previously in Taiwan, the National Health Insurance has allowed the use of cemented stems only for BHA surgery for femoral neck fracture patients aged over 70 years. However, a good outcome using cemented stems relies on great cementing technique, which inexperienced young surgeons may struggle with during surgery. For modern cementless hip stems, advancement of the surface coating technique with a hydroxyapatite layer enhanced osteointegration for better fixation efficiency at the bone–implant interface. A clinical report by Figved *et al*.^16^ and Sköldenberg *et al*.^17^ supports the usage of hydroxyapatite‐coated hip stems for cases of femoral neck fracture. In the current study, 43 patients aged over 70 years who suffered from femoral neck fractures underwent BHA surgery with cementless hip stems. Only 1 of the 43 cases underwent revision surgery due to infection. No stem loosening or other stem‐related complication was found. Our results also support that using cementless stems with hydroxyapatite coating is an acceptable means of treatment for femoral neck fractures in elderly populations. For young surgeons who may not be experienced in cementing technique, using cementless hip stems with hydroxyapatite coating is an alternative for patients with fractured femoral necks.

Some limitations of the current study should be noted. Due to the lack of information on the preoperative status of patients enrolled in the current study, comparison for improvement after surgery is not possible. Besides, the number of revision cases reported was low on the current study; thus, we cannot use statistical methods to evaluate the major influence on prosthetic revision. Finally, long‐term review over 10 years is quite difficult due to insufficient numbers of subjects remaining.

### *Conclusion*

In the current study, both THA and BHA for the treatment of hip disorders revealed high survival rates at 10‐year follow up, with identical cementless femoral stems implanted. Problems relating to leg length discrepancy and preoperative planning for avascular necrosis cases must be addressed, while techniques for pain control or avoidance should be considered for BHA in further studies and in the process of implant development.
